# Applications of Micro-Indentation Technology to Estimate Fracture Toughness of Shale

**DOI:** 10.3390/ma13184208

**Published:** 2020-09-22

**Authors:** Qiang Han, Zhan Qu, Ping Wang, Gang Bi, Guanzheng Qu

**Affiliations:** 1 College of petroleum engineering, Xi’an Shiyou University, Xi’an 710065, China; zhqu@xsyu.edu.cn (Z.Q.); wp8230@xsyu.edu.cn (P.W.); big@xsyu.edu.cn (G.B.); quguanzheng@xsyu.edu.cn (G.Q.); 2 Shaanxi Key Laboratory of Well Stability and Fluid & Rock Mechanics in Oil and Gas Reservoirs, Xi’an Shiyou University, Xi’an 710065, China

**Keywords:** shale, micro-indentation, mechanical property, fracture toughness

## Abstract

The fracture toughness of shale is a basic parameter that can provide effective theoretical support for wellbore stability and hydraulic fracturing of a shale reservoir. Due to the composition and microstructure, there are many problems in evaluating the mechanical properties of shale in a macroscopic test. In this paper, the composition and pore distribution of shale were studied by X-ray diffraction and nuclear magnetic resonance. Scanning electron microscopy was used to characterize the pore structure. The setting of experimental parameters and the selection of the indenter were discussed. Micro-indentation technique was proposed and applied to fracture toughness analysis of shale. The results show that Berkovich indenter is more suitable for shale indentation test than Vickers indenter. Fracture toughness of shale indentation is obviously affected by surface roughness and indentation position. Fracture toughness of shale decreases slightly with the increase of the indentation load. The energy analysis result presents that the effect of cracking on the ratio of total/unloading work is minimal when there is no significant stripping on the shale surface. Compared with the experimental method, energy methods can obtain all the analysis parameters from a single indentation test. The results of comparative analysis with macroscopic experiments display that micro-indentation test can effectively predict the macroscopic fracture toughness of shale.

## 1. Introduction

As a basic technical parameter, the fracture toughness of shale can provide effective theoretical support for wellbore stability and hydraulic fracturing of shale reservoir [1]. Though there is plenty of research on rock fracture, they remain with the fields of the macro experiment and the corresponding constitutive relation. The macro-mechanics experiment has some problems, such as large sample size, time consuming and low interpretation accuracy [2]. The method of establishing fracture prediction model by logging interpretation with downhole instruments is still limited by downhole construction [3]. It is of scientific significance and practical value to acquire shale fracture characteristics in time at the early stage of development, which can effectively control wellbore instability and hydraulic fracturing in the construction process.

With the development of material preparation, instrumented indentation technology has been applied in many fields gradually [4,5]. This technology not only can test material’s elasticity, hardness, fracture initial failure information, but also indirectly evaluate a variety of mechanical response combined by dimensional analysis. The technique is divided into micro/nano-indentation test according to the indentation displacement scale. The performance evaluation of brittle materials based on instrumental indentation test has been explored for half a century. Based on the fracture mechanics theory and micromechanical experimental observation, some models have been constructed for the analysis of microscopic fracture toughness [6,7]. Typical fracture toughness testing methods can be classified according to the geometry shape of the indenter [8]. The indenter shapes used in the test mainly include pyramidal and spherical shapes [9,10]. Pyramid indenter has a merit that detection of crack length is relatively accurate and a demerit that the analysis of elastic-plastic deformation field is complex. For a spherical indenter, the advantage is that the analysis of the elastic deformation field is simple, but it also has the disadvantages of crack measurement accuracy. In terms of the theoretical research, the method of measuring fracture toughness with a Vickers indenter under the condition of full crack propagation is established based on the Lawn–Evans–Marshall model [11]. The uniform expression of Vickers fracture toughness is established by indentation tests on shot diameter crack and fully extended crack [12]. The fracture toughness of Berkovich indenter is obtained by stress intensity factor model of star crack in thick-walled cylinder under internal pressure [13]. Another way to evaluate fracture toughness first proposed by Cheng is scaling relationship of indentation work, which is based on dimensional analysis and finite element simulation [14]. The scaling relationship of indentation work is established by the using the hole expansion model. In this method, total energy and unloading energy are used to replace the measurement parameters dependent on indentation test. The scaling relationships of reduced modulus, hardness and energy were evaluated by experimental verification and theoretical analysis. By analyzing the change of indentation fracture work, Liu et al. investigated the relationship between fracture energy and fracture toughness [15]. The relationships of total energy, fracture energy and irreversible energy were further discussed, and the solution of fracture toughness was optimized. Instrumented indentation technology for fracture toughness has been extensively studied and got some achievements on biomaterials, hard tissue and crystalline silicon [16,17].

With the in-depth study of heterogeneous materials, instrumented indentation technology has been gradually introduced into the study of shale mechanics in the early 21st century. The mechanical properties of nano-scale porous clay particles were evaluated. Statistical methods of indentation mechanics for nanocomposites (concrete, shale) were established [18,19]. Shale is a multi-component rock that contains nano-scale porosity, clay particles, non-clay inclusions and perhaps even a small amount of kerogen [2]. The mechanical properties of shale are related to its composition and micro-pores. According to the multi-scale composition character, the mechanical scale of shale consists of nano-, micro- and macro-scale. Nano-scale refers to a scale on the order of 10^−8^ to 10^−6^ m, and focuses on clay minerals and inter-granular pores. Micro-scale refers to a scale on the order of 10^−5^ to 10^−4^ m. and focuses on porous clay and inclusions [20]. Macro-scale is considered at the scale of 10^−3^ m and above. Han Qiang et al. carried out the evaluation of shale elastic modulus, indentation hardness, analyzed the influencing factors of the experiment, and provided guidance for the prediction of shale macroscopic mechanical properties [21,22,23]. Shale has been mechanically studied at the nano-scale by researchers in China and elsewhere, and although a number of valuable evaluations have been made, these are still insufficient. At present, the study on shale fracture toughness analysis at micro-scale test is less. The task of micromechanics is to find the properties of macro-scale materials based on the information of microstructure. It is clear to see that micro-scale research is of great importance as a bridge between the nano- and macro-scale. In this paper, applications of micro-indentation technology to estimate fracture toughness of shale were performed and analyzed. Influence factors of micro fracture of shale were estimated from the shape of indenter, effect of cracking and effective evaluation method. The shale fracture toughness of micro-indentation was evaluated and the macro-fracture toughness was predicted.

## 2. Samples Preparation

**Figure 1:**
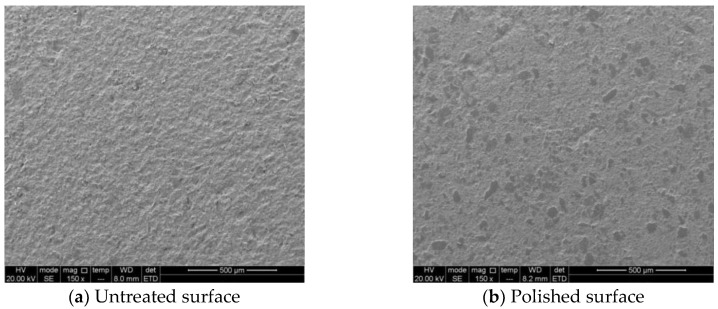
The images of shale surface treatment.

The test samples were taken from the outcrop of Longmaxi formation shale in Sichuan basin in China. Based on the X-ray diffraction (XRD), the mineral compositions of samples were derived. Nuclear magnetic resonance (NMR) was used to study the pore distribution of shale. The microstructure and composition of samples were observed by Scanning electron microscopy (SEM). The indentation test sample size was 50 × 25 × 15 mm. According to the characteristics and test requirements, shale surface was polished to reduce the effect of surface roughness on indentation test [21]. The first step was a coarse grinding. Sandpaper with different sizes of grit from 400 to 1200 was used to grind the sample surface. The second step is the fine polishing. Diamond polisher was used to polish the shale sample to the desired smoothness. Figure 1 shows the shale surface morphology on untreated and polished surfaces.

## 3. Micro-Indentation Tests

### 3.1. Equipment

Micro-indentation test for this study was performed on the test platform MFT-4000. The platform integrates a variety of technologies such as acoustic emission detection, friction detection, micro-scale displacement measurement and microcomputer automatic control. The apparatus is capable of applying and measurement loads between 0 and 300 N with a resolution of 0.5 N. The loading rate is 5–100 N/min. The maximum indentation depth is 200 μm with a resolution of 0.5 μm. A series of indentation calibrations has to be performed before the test begins. The load and indentation depth transducer are calibrated by using friction signal processor, load magnification potentiometer and zero potentiometer. Another calibration is performed when apparent load is inconsistent with the added weight. The potentiometer is linearly adjusted until the weight is consistent with the amplifier’s data. The abnormal section on the curve can be selected and amended in the optimization module.

Another vital component of the indentation test is indenter, which can be classified as pyramid indenter (Vickers indenter and Berkovich indenter), conical indenter, spherical indenter and cylindrical indenter. The spherical indenter is appropriate for measuring the continuous change of indentation strain. The cylinder indenter is suitable for measuring large initial contact stiffness material. The Vickers indenter, a four-sided pyramid, is a standard indenter used for micro-hardness testing. Sometimes, the geometrical shape of the Vickers indenter cannot be self-similar under different scale because of the error of machining accuracy. This phenomenon may introduce errors in following study, affect measurement accuracy in instrumental indentation test. In order to address this problem, Berkovich indenter, with the equivalent parameters of a Vickers indenter, is designed. For shale, the Vickers and Berkovich indenters were utilized in this study.

### 3.2. Typical Procedures

**Figure 2:**
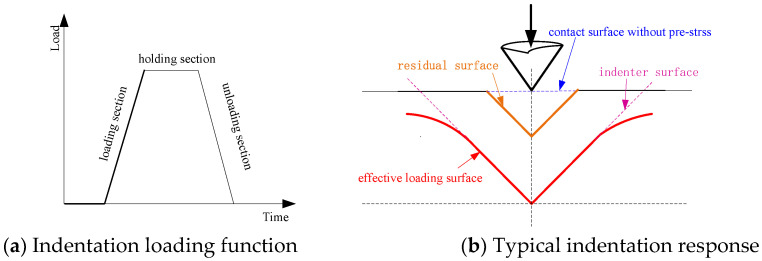
Typical indentation loading function and indentation change schematic diagram.

The general procedure for an indentation test consists of several steps (Figure 2). First, the indenter tip was in contact with the sample surface without prestress. Next, calibration was performed to effectively reduce the systematic errors in the test data. Once indentation begins, the indenter tip was loaded on the sample surface at a constant loading rate. When the maximum load was obtained, this load was held for 15 s to allow the sample to undergo time-dependent deformation. Finally, the indenter tip was retracted from the sample surface at a constant rate. During the experimental process, the load and depth data of the indenter were recorded by the sensor.

### 3.3. Parameter Setting

Since shale is a multiphase composite rock, a random indentation cannot sample the overall mechanical response of shale. The Delesse principle shows that the surface fraction of a disordered material is equivalent to the volume fractions [24]. Therefore, the overall mechanical response of the shale can be shown by gridded indentation analysis. ISO 14577 states that the distance between adjacent indentations is at least 10 times of the indentation diameter. Based on the standard and actual test results of shale, the distance between two indentations was 2 mm, and the indentation was 9 mm from the sample boundary [23]. To determine the indentation load to shale at the micro-scale, a large number of indentation experiments of shale were performed. When the maximum load is 60 N, the sample surface is damaged and the cracks appeared.

### 3.4. Environmental Control

As an important external influence factor, the experimental environment has to be controlled in the experiment. The material surface and instruments may expand or contract due to temperature fluctuations in the indentation test. This phenomenon can influence the measuring accuracy of the indentation depth. The test should be performed at the specified temperature and humidity range. It was stipulated that the temperature of shale micro-indentation test was 25 ± 1 °C, and the relative humidity was lower than 50%.

Another external influence factor is ground vibration. The amplitude of ground vibration is generally in the micro-scale. To reduce the impact of ground vibration on the indentation depth, the module of shock absorber was placed on MFT-4000 tester. In addition, the test module of the instrument was placed on the experimental platform with fixed function. These measures can effectively reduce the vibration caused by the surrounding environment.

## 4. Determination of Fracture Toughness by Indentation Test

### 4.1. Experimental Analysis

The fracture toughness of micro-scale brittle materials can be evaluated by micro-indentation test. According to the stress field of indenter acting on the material surface and the test of the geometry size of the crack, the computation model is constructed to solve the fracture toughness. This method only needs to ensure that the surface of the sample is smooth plane, and it has almost no limit on the shape and size. The indenters commonly used for the fracture study of brittle materials are Vickers and Berkovich indenters. The typical experimental method of fracture toughness is discussed in this part.

(1) Vickers indenter

According to the elastic/plastic indentation damage model, indentation area is composed of semi-spherical plastic zone and elastic zone around the periphery. It is assumed that the radial crack on the sample surface generated by the indentation corresponds to half-penny crack that penetrates inside the sample [11]. In this case, elastic stress field during loading stage is divided into the recovery part and the unrecoverable part of the plastic deformation during unloading stage. The recovery part inhibits the radial expansion of the half-penny crack surface. In contrast, the unrecoverable part may promote the radial expansion of the crack [25]. Therefore, half-penny crack can be further extended along the sample surface during the unloading. Elastic/plastic indentation damage model provides a way to evaluate fracture toughness using Vickers indenter in a condition of sufficient crack propagation. Under the condition of full crack growth, the elastic–plastic deformation interaction can be simplified as a pair of concentrated forces perpendicular to the center of the half-penny crack surface. Based on the analytical solution of embedded disk stress intensity factor [26], the stress intensity factor at the front of half-penny crack can be expressed as:

(1) $$K_{r}\infty f\left(\phi_{v}\right)\frac{F_{r}}{c^{3/2}}=f\left(\phi \right){\left(\frac{E}{H}\right)}^{1/2}{\left(\cot\psi \right)}^{3/2}\frac{F_{m}}{c^{3/2}}$$

where *K_r_* is the stress intensity factor; *F_r_* and *F_m_* are a pair of concentrated forces; *E* is the elastic modulus; *H* is hardness; *Ψ* is half angle of the indenter opposite edge; *f*(*φ*) is a correction factor of stress intensity factor. When *φ* = π/2, crack propagation equilibrium condition along the radial direction is given by

(2) $$K_{IC}={K_{r}|}_{\phi =\pi /2}$$

Thus, the fracture toughness is given by [27]

(3) $$K_{IC}=\delta {\left(\frac{E}{H}\right)}^{1/2}\frac{F_{m}}{c^{3/2}}$$

where *δ* is the coefficient, determined by experimentation, related to the geometry of the indenter.

For some brittle materials, radial crack is dominant under the action of Vickers indenter. In this case, it is inaccurate to obtain fracture toughness using Equation (3). Short radial crack often occurs in the case of low indentation loading or superior toughness of the material. For a short radial crack, the effect of the plastic zone on the peripheral zone cannot be reduced to point load. For this reason, the study of fracture toughness is composed of short radial crack and fully expanded crack. Based on the experimental analysis, the uniform expression of fracture toughness for short radial crack and fully extended radial crack can be written as [28]

(4) $$14\left[1-8{\left(\frac{4v-0.5}{1+v}\right)}^{4}\right]\left(\frac{K_{IC}\Phi }{H\sqrt{a}}\right){\left(\frac{1}{\Phi }\frac{H}{E}\right)}^{2/5}={\left(\frac{c}{a}\right)}^{c/18a-1.51}$$

(2) Berkovich indenter

**Figure 3:**
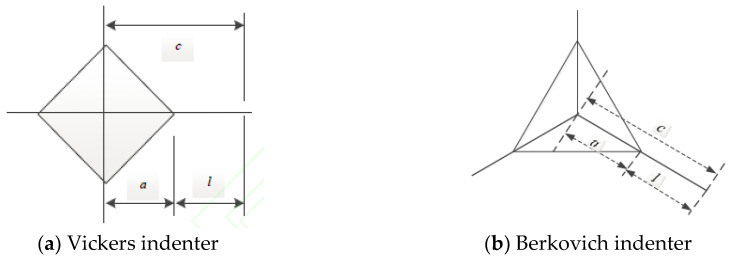
Schematic diagram of radial crack with two indenters.

The appearance of Berkovich indenter is to eliminate the influence of the transverse edge of Vickers indenter on the test. The two indenters have the same surface area at same indentation depth. The difference between the two indenters is the number of lateral edges. Based on the study of the expansion loaded star crack, the difference between two indenters in determining fracture toughness has been studied [29]. In the case of fully extended crack, the internal crack morphology of the material has little effect on the fracture toughness. The fracture toughness of Berkovich indenter is expressed as [29]

(5) $$K_{IC}=1.073\delta^{L}{\left(\frac{a}{l}\right)}^{1/2}{\left(\frac{E}{H}\right)}^{2/3}\frac{F_{m}}{c^{3/2}}$$

where *δ^L^* is the coefficient related to Berkovich indenter. Figure 3 show the schematic diagram of radial crack with Vickers and Berkovich indenter, where *a* is indentation radius, *c* is the length of radial crack, *l* is the length between indentation and crack tip.

Regardless of the type of indenter, Equations (4) and (5) show that the elastic modulus and hardness need to be determined by other way. The Oliver-Pharr principle, based on elastic theory, is a typical analysis method to obtain elastic modulus and hardness [23]. This mechanical mechanism of the Oliver-Pharr analysis is clear and the calculation is simple and feasible. The elastic modulus is written as [27]

(6) $$E=\frac{1-\nu^{2}}{\frac{1}{E_{r}}-\frac{1-\nu_{i}^{2}}{E_{i}}}$$

where *E* is the elastic modulus of test sample, *E_r_* is the reduced modulus of indentation, *E_i_* is the elastic modulus of indenter, *v* is poisson’s ratio of sample, and *v_i_* is poisson’s ratio of indenter.

Hardness is a functional index of resistance to external force. Hardness is related to the elastic/plastic deformation and measurement method of material, defined as [27]

(7) $$H=\frac{F_{m}}{A_{c}}$$

where *H* is the hardness; *A_c_* is indenter area function related to indentation displacement. It should be noted that *E* and *H* are derived from the indentation test where the material surface is not broken.

### 4.2. Energy Analysis

(1) Total/unloading energy

**Figure 4:**
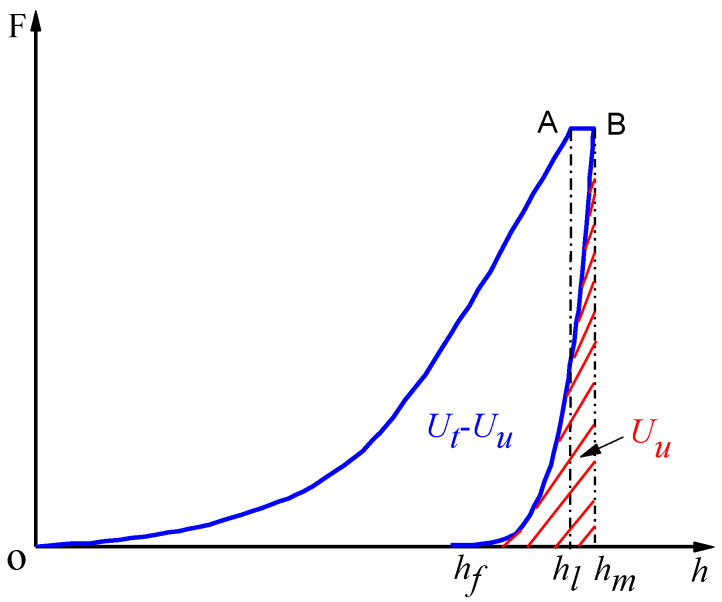
Schematic illustration of shale micro-indentation load-displacement curve.

For some materials such as composite metal or heterogeneous media, there are some defects in the experimental analysis. Not all parameters such as *E* and *H* can be measured by a single indentation fracture test. In order to solve this problem, the change of energy during indentation is studied. According to the energy scaling relationship of indentation, the ratio of *E*/*H* in Equations (3) and (5) can be replaced with the ratio of total/unloading energy [30]. Figure 4 shows the schematic illustration of shale micro-indentation load–displacement curve.

The area integral of the underlined part in red is unloading energy, *U_u_*. The area integral of the curve OABh_m_ is total energy, *U_t_*. In the case of uncracking, the approximate linear relationship is given by [30]

(8) $$\frac{H}{E_{r}}\approx \kappa \frac{U_{u}}{U_{t}}$$

where *κ* is a dimensionless constant related to the shape of the indenter. Based on indentation stress-strain flied analysis, the relationship between *H*/*E_r_* and *U_u_*/*U_t_* can be determined as [31,32]

(9) $$\frac{H}{E_{r}}=\frac{2\left(1-\nu \right)}{3}\cot\beta \cdot \frac{U_{u}}{U_{t}}$$

where *β* is equivalent semiconical angle. Inserting Equations (8) and (9) into Equation (3), fracture toughness can be expressed as

(10) $$K_{IC}=\lambda {\left(\frac{U_{u}}{U_{t}}\right)}^{-1/2}\frac{F_{m}}{c^{3/2}}$$

where *λ* is dimensionless constant determined by the indenter geometry. It is worth noting that the application condition of total/unloading energy method is that the effect of cracking on the ratio of *U_u_*/*U_t_* can be ignored.

(2) Fracture energy

In the conventional mechanical experiment, residual strain can be retained on the surface of the material and the energy is released completely after unloading. The theory of fracture energy states that the total stored energy is partially released during unloading and the rest of the energy is stored. The stored energy below the residual micro-indentation cannot be released under loading condition. This is due to residual indentation and its periphery is not affected by the force boundary. This part of the stored elastic energy and the plastic dissipation energy should be treated differently because it can be released under certain condition such as fracture. Therefore, total energy can be partitioned into three typical components: elastic, plastic and fracture energy. Total energy and elastic energy can be calculated from the indentation curve. Fracture energy is given by [33]

(11) $$U_{crack}=U_{ir}-U_{p}$$

(12) $$U_{ir}=U_{t}-U_{u}$$

where *U_p_* is the energy consumption due to pure plasticity; *U_ir_* is irreversible energy. *U_p_* is an important parameter to calculate fracture energy. Based on the analysis of plastic and total energy, the ratio of *U_p_*/*U_t_* is written as [33]

(13) $$\frac{U_{p}}{U_{t}}=1-\frac{(1+n)\left(1-\frac{h_{f}}{h_{m}}\right)}{\left(1+m)(1+n-n\frac{h_{l}}{h_{m}}\right)}$$

where *h_f_* is the residual displacement of indenter; *h_m_* is the maximum displacement of indenter; *h_l_* is the maximum load phase displacement; *n* and *m* are the power indexes for the loading and unloading indentation curves (Figure 3). The power function of a curve is elicited by fitting the load-displacement curve. And then *U_u_* and *U_t_* are obtained by the definite integral computation of load-displacement curve. The fracture energy per unit area is defined as the critical energy release state. Therefore, the fracture toughness can be written as [34].

(14) $$K_{IC}=\sqrt{\frac{U_{crack}}{A_{c}}\cdot E_{r}}$$

## 5. Results and Analysis

### 5.1. Mineral Composition and Pore Structure

The mineral composition of shale was analyzed by X-ray diffraction experiment. Quantitative analysis displays that quartz and clay are the two main mineral constituents in shale samples. Additionally, the samples contain small amounts of calcite, dolomite, pyrite, potassium feldspar and plagioclase (Table 1). The results show that samples 1 and 2 have similar clay minerals and quartz. Sample 3 has the most clay minerals and quartz. The total content of clay minerals and quartz is 46.3–86.4%. It can be seen that the shale in Longmaxi Formation is mainly composed of medium-hardness minerals. The samples are prone to brittle failure due to the high content of medium brittle minerals.

**Figure 5:**
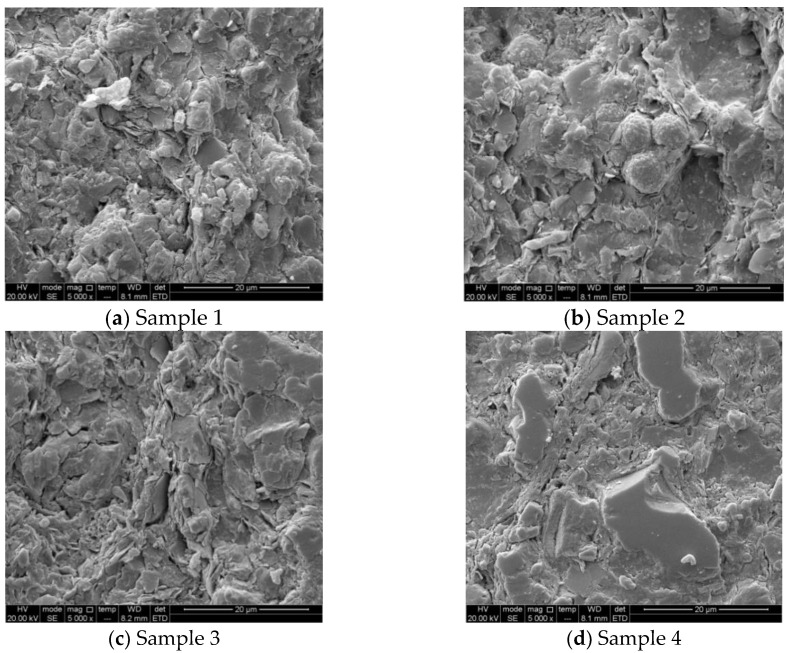
SEM images of four samples.

Nuclear magnetic resonance (NMR) was used to study the pore distribution of shale. Figure 5 presents the scanning electron microscopy (SEM) images of four samples. The bedding and micro-crack are well-developed. Clay and non-clay minerals can be observed directly. The surface structure of sample is compact, and flake clay minerals and insoluble residues can be seen. Non-clay minerals are evident, and authigenic carbonate minerals, pyrite, feldspar are developed on the surface. This microstructural characteristic of shale may lead to the concentration of tectonic stress or the intensification of microcracks after hydration, resulting in the fracture and spalling of the rock in the wellbore.

**Figure 6:**
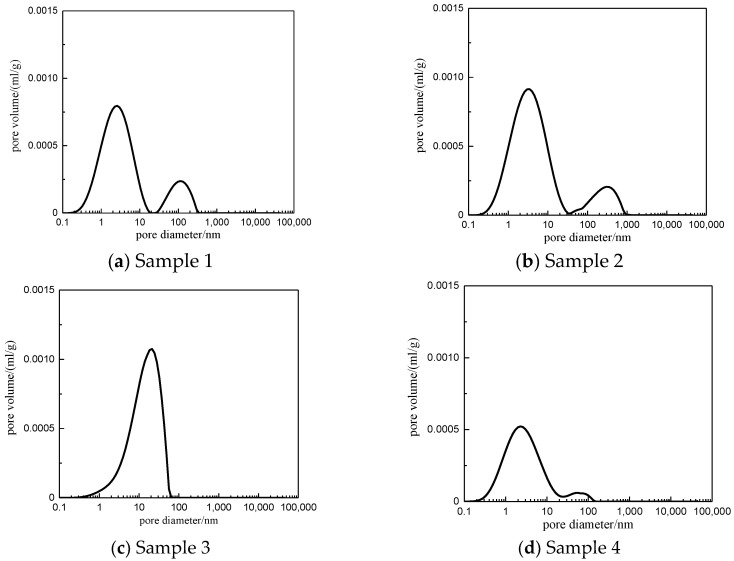
The pore size distribution of shale results from the NMR test.

Figure 6. displays the pore size distribution of four samples. It is seen that sample 1 and 2 have, in order, similar pore distribution. The pore size of four samples is 0.05–120 nm. The left peak in Figure 6 is distributed symmetrically and has a large peak, which indicates the development of microporous to mesoporous samples.

### 5.2. Micro-Indentation Test

**Figure 7:**
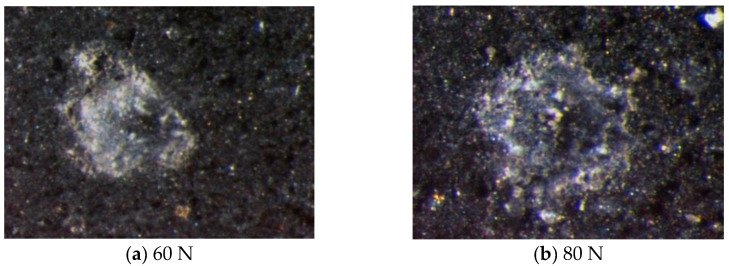
Typical micro-indentation images of shale by using Vickers indenter.

Figure 7 depicts typical micro-indentation of shale obtained by the Vickers indenter. The maximum loads are 50 and 80 N. It is observed that the Vickers indenter resulted in small pits, small drop-cuts and exfoliation on the surface of shale micro-indentation. The self-similarity between Vickers indenter and shale micro-indentation is not satisfactory. One of the key difficulties in the indentation analysis is the determination of the contact area. The contact area is irregular and there is no obvious radial crack on the shale surface (Figure 7). In addition, the load–depth curve appears as irregular bump. Thus, it is difficult to obtain the length of radial crack and indentation semi-diagonal. The main reason for this phenomenon is the chisel edge structure of the Vickers indenter tip and the heterogeneous composition of shale.

**Figure 8:**
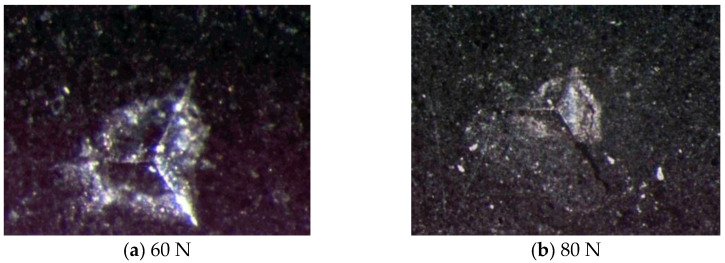
Typical micro-indentation tomography of shale by using Berkovich indenter.

In order to get an accurate assessment, we also performed indentation by using Berkovich indenter. Maximum loads are 60 and 80 N. Figure 8 presents the typical micro-indentation of shale obtained by Berkovich indenter. It is seen that the similarity between Berkovich indenter and indentation is better than that of Vickers indenter. The surface crack appeared when the load reached the threshold of shale fracture. It is observed that surface radial crack began to develop at 60 N. The crack development is obvious when the maximum load is 80 N. According to the previous analysis (Section 4.1), the length of radial crack is the important parameter. It is clear that Berkovich indenter is suitable for shale fracture evaluation.

**Figure 9:**
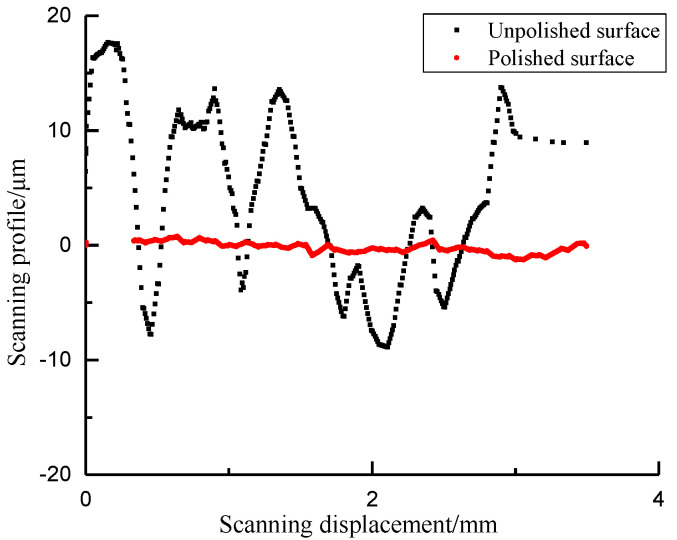
Shale surface roughness.

**Figure 10:**
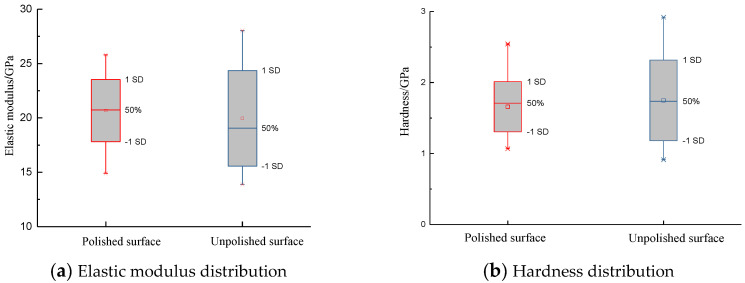
The relationship between surface properties and mechanical parameters.

Shale is composed of granular minerals with a certain roughness on its surface. The surface roughness is related to the indentation displacement and contact area. If the characteristic length of the roughness is the same magnitude as the indentation displacement, the contact area obtained at the sink-in point of the shale surface is lower than the true value and higher than the true value at the pile-up point. Therefore, it is suggested that the displacement length of indentation test is no less than 20 times the surface roughness. It can be ensured that the uncertainty of indentation displacement caused by surface roughness is less than 5%. Figure 9 compares the results of surface scanning profile before and after mechanical polishing. The scan profile displacement of unpolished surface varies from −10 to 19 μm. Surface toughness has obvious influence on the elastic contact displacement. The scan contour displacement of polished surface decreases obviously, and the contact area function is less affected. Elastic modulus and hardness can be greatly affected by fluctuating displacement. In the experimental analysis, elastic modulus and hardness are important parameters to obtain fracture toughness. Figure 10 shows the distribution of elastic modulus and hardness before and after surface polishing of shale by micro-indentation test. The measured elastic modulus and hardness of the polished surface converge to those of the unpolished surface. It is necessary to polish the sample surface before indentation test.

**Figure 11:**
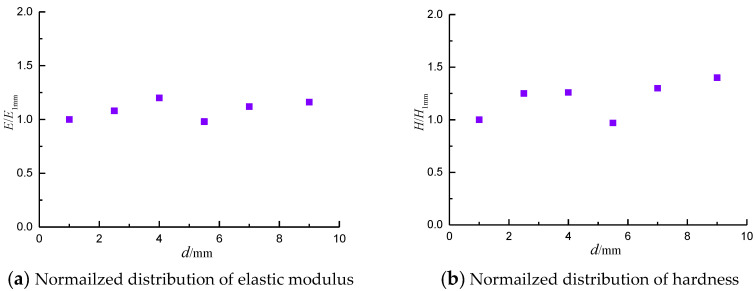
The effect of the indentation position on the indentation test.

It is found that the boundary effect is obvious when the micro-indentation position is too close to the edge of the shale. Figure 11 displays the distribution between normalized mechanical parameters (elastic modulus, hardness) and boundary, where *E*_1mm_ and *H*_1mm_ are the elastic modulus and hardness at 1 mm from the edge of the shale surface. It is clear that the elastic modulus and hardness of indentation are relatively stable at 4 mm away from the boundary. A suitable indentation position facilitates the solution of fracture toughness.

### 5.3. Fracture Toughness

**Figure 12:**
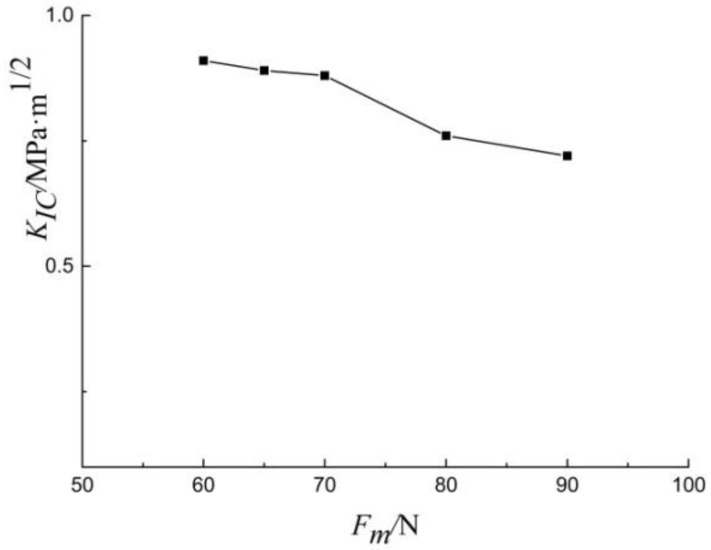
*K_IC_* versus *F_m_* generated with the Berkovich indenter.

Length of radial crack and radius of micro-indentation are two important measurement parameters for fracture toughness indentation test. Figure 12 displays the relationship between *K_ic_* and *F_m_*. It is clear that *K_ic_* decreased slightly with the increase of load. The crack lengths for shale samples are 1–3 mm for Berkovich indenter. These small cracks which are difficult to measure can cause measurement deviation of fracture toughness. As previously discussed, compared with Vickers indenter, the geometry of Berkovich indenter is more conducive to a sharp tip. Tip geometry has a critical effect on fracture initiation.

**Figure 13:**
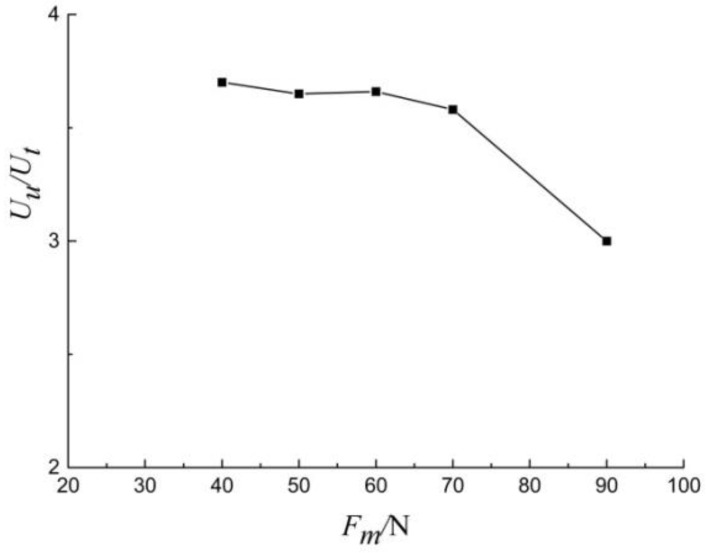
*U_u_*/*U_t_* versus *F_m_* generated with the Berkovich indenter.

The ratio of *U_u_*/*U_t_*, which is easy to measure, is taken as the fundamental parameters in energy analysis. This method can determine all the analytical parameters from a single indentation test and simplify the test process. Since the analytical model utilizes the uncracked indentation energy scale relationship, it is necessary to satisfy the condition that the effect of cracking on *U_u_*/*U_t_* can be ignored. Therefore, the applicability of this condition to shale needs to be discussed. For shale, the critical load for cracking is lower, and the shale is susceptible to cracking by using Berkovich indenter. Figure 13 displays the relationship between *U_u_*/*U_t_* and indentation load. Maximum indentation loads were 40, 50, 60, 70 and 90 N. It is clear that crack is fully expanded without surface peeling when the indentation load is less than 70 N. The ratio of *U_u_*/*U_t_* is generally stable with the increase of the indentation load. When indentation load is more than 70 N, the ratio of *U_u_*/*U_t_* decreases gradually with the increasing load. The main reason is the indentation surface peeling off. Experimental results showed that if there is no obvious material peeling, the effect of cracking on *U_u_*/*U_t_* is not significant even if radial cracks are fully extended. When there is obvious material peeling on the indentation surface, the ratio of *U_u_*/*U_t_* is affected significantly and cannot be ignored.

**Figure 14:**
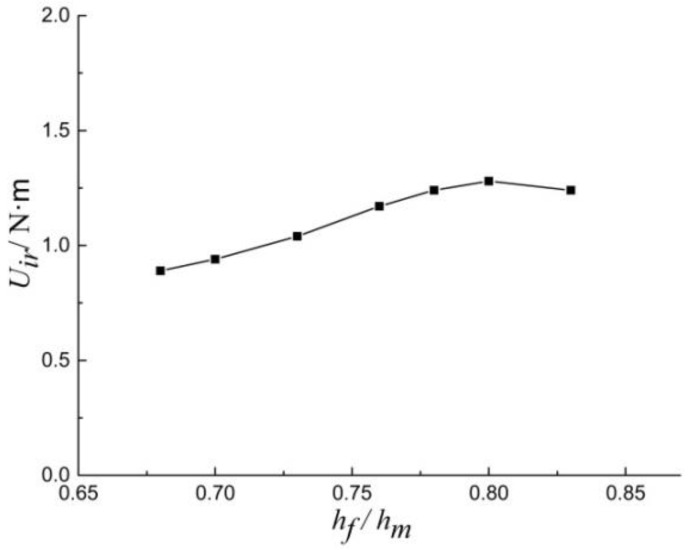
*U_ir_* versus *h_f_*/*h_m_* generated with the Berkovich indenter.

Analysis of fracture energy provides a way to evaluate the relationship between fracture energy and fracture toughness. Figure 14 compares the *U_ir_* with the ratio of *h_f_*/*h_m_*. It is concluded that when *h_f_*/*h_m_* is between 0.68 and 0.84, *U_ir_* differ by 0.1~0.4 N·m, and the *U_ir_* value shows an upward trend.

**Figure 15:**
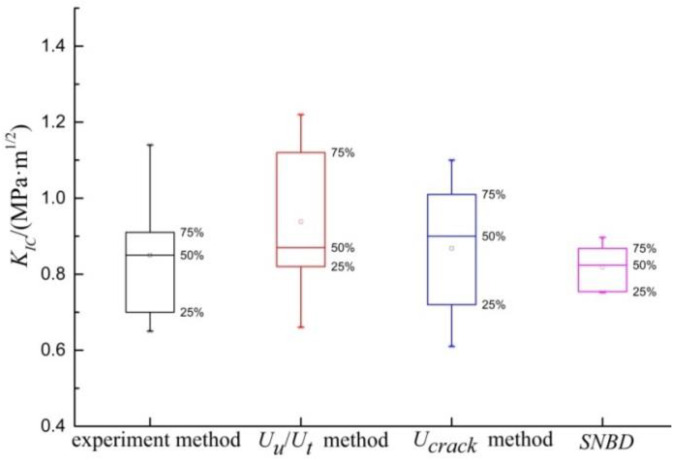
Comparative analysis of shale fracture toughness.

The results of straight-notched Brazilian disk (SNBD) show that the fracture toughness of shale at macro-scale is about 0.82 MPa·m^1/2^ [35]. In this paper, the SNBD results are considered as agreed truth values of shale fracture toughness. These agreed truth values are used to compare the reliability of the analysis micro-indentation test. The indentation load in the test is taken as 70 N. Figure 15 shows the fracture toughness distribution by micro-indentation experiment, total/unloading energy method, fracture energy method and SNBD. The *K_IC_* of experiment method is 0.65–1.14 MPa·m^1/2^, 0.86 MPa·m^1/2^ on average. The distribution deviation from 25–75% is less than 0.22. The *K_IC_* of *U_u_*/*U_t_* method is 0.66–1.22, 0.94 MPa·m^1/2^ on average. The distribution deviation from 25–75% is less than 0.32. The *K_IC_* of *U_crack_* method is 0.72–1.1, 0.87 MPa·m^1/2^ on average. The distribution deviation from 25–75% is less than 0.31. It is found that the average *K_IC_* of experiment method accord well with the SNBD results. When experimental parameters are not easy to obtain, energy method can be as a simplified approximate solution.

## 6. Conclusions

In this paper, shale mineral composition and microstructure were analyzed from the outcrop of Longmaxi Formation. Fracture experiments of shale micro-indentation were carried out by using Vickers indenter and Berkovich indenter. The basic characteristics of mesoscopic fracture toughness of shale were analyzed. The key results can be summarized as follows.

(1) XDR results show that shale of Longmaxi Formation is prone to brittle failure due to the high content of medium brittle minerals. The content of clay minerals and quartz is 47.8–86.4%. NMR analysis displayed that shale of Longmaxi Formation pores microstructure is extremely developed. The surface structure of sample is compact, and flake clay minerals and insoluble residues can be seen.
(2) Based on the shale micro-indentation test, Vickers and Berkovich indenters are evaluated. The self-similarity between Vickers indenter and shale micro-indentation is not satisfactory. For the Berkovich indenter, the crack development is obvious when the load reaches 80 N. The results show that the Berkovich indenter can be used for micro-indentation testing of shale fracture toughness. The relationship between Length of radial crack and radius of micro-indentation is approximately linear. The fracture toughness of shale decreases slightly with the increase of indentation load. The problem with this method is that multiple indentation experiments are needed because the intermediate parameters cannot be measured in the same test.
(3) Energy analysis methods are introduced to determine all parameters from a single micro-indentation test. The results show that the effect of cracking on the ratio of *U_u_*/*U_t_* is minimal when there is no significant stripping on the shale surface. *U_u_*/*U_t_* energy method can be used to evaluate the fracture toughness by replacing the ratio of *E/H*. It is clear that the ratio of *U_u_*/*U_t_* decreases gradually with the increase of the indentation load. The fracture energy method provides a way to evaluate the relationship between fracture energy and fracture toughness. Furthermore, the analysis and comparison of the results demonstrate that the average *K_IC_* of experiment method accord well with the SNBD results. When experimental parameters are not easy to obtain, the energy method can be used as a simplified approximate solution. The present work provides a novel idea regarding the macroscopic prediction of fracture behavior of shale.

## Figures and Tables

**Table 1:** Total mineral composition of shale in the Longmaxi Formation.

Sample No.	Mass Percentages of Shale/%						
	Clay	Quartz	Orthoclase	Plagioclase	Calcite	Dolomite	Pyrite
1	33.6	17.8	2.1	3.7	32.9	9.3	0.6
2	32.1	14.2	1.6	2.1	25.4	2.3	22.3
3	48.3	38.1	0.8	6.4	2.8	2.4	1.2
4	52.3	30.2	2.5	3.7	4.2	6.0	1.1
